# Oncolytic immunotherapy for the treatment of non-muscle invasive bladder cancer using intravesical coxsackievirus A21

**DOI:** 10.1186/2051-1426-3-S2-P331

**Published:** 2015-11-04

**Authors:** Nicola E Annels, Guy Simpson, Mehreen Arif, Mick Denyer, Attya Iqbal, David Mansfield, Sarbjinder Sandhu, Alan Melcher, Kevin Harrington, Gough Au, Mark Grose, Darren Shafren, Hugh Mostafid, Hardev Pandha

**Affiliations:** 1 University of Surrey, Guildford, UK; 2 The Institute of Cancer Research, London, UK; 3 University of surrey, Guildford, UK; 4 St. James's Institute of Oncology, St. James's University Hospital, Leeds, UK; 5 Institute of Cancer Research and Royal Marsden Hospital, London, UK; 6 The University of Newcastle, Newcastle, Australia; 7 Viralytics Limited, Toronto, ON, Canada; 8 Viralytics, Sydney, Australia

## Background

As a clinical setting in which local live biological therapy is already well established, nonmuscle invasive bladder cancer (NMIBC) presents intriguing opportunities for oncolytic virotherapy. Coxsackievirus A21 (CVA21) is a novel intercellular adhesion molecule-1 (ICAM-1) targeted immunotherapeutic virus. Combining CVA21 with low doses of chemotherapy (Mitomycin C) enhances CVA21 viral replication and oncolysis by increasing expression levels of ICAM1 on bladder cancer cells. As well as initiating oncolysis, oncolytic viruses can also induce immunogenic cell death (ICD) determinants which is thought to be the optimal way to trigger a tumour-specific response and crucial for long-term therapeutic success. This study set out to confirm the enhanced cytotoxicity in vitro of bladder cancer cell lines and tissues following combined treatment of mitomycin C and CVA21 and to demonstrate the induction of immunogenic cell death determinants.

## Methods

Characterization of CVA21 cytotoxicity in a panel of 6 bladder cancer cell lines yielded a range of sensitivities. CVA21 cytotoxicity largely correlated with expression of the viral receptor ICAM-1. CAV21 in combination with Mitomycin C resulted in up-regulation of ICAM-1 and significantly increased cytotoxicity over CVA21 alone. Furthermore, CVA21 induced the expression of multiple ICD determinants (calreticulin, ATP and HMGB1 release) and the immunogenic cell marker, Fas, associated with susceptibility to immune attack. The expression of these immunogenic markers was up-regulated in the CVA21 and combination treated cells as compared to Mitomycin C only and untreated cells. The caspase-glow 3/7 assay revealed apoptosis as the primary mode of death. This was further confirmed with the use of an apoptotic inhibitor (Z-VAD-FMK) which significantly inhibited cell-death of CVA21 infected cells.

## Results

The above data provided the rationale for a two stage Phase I study in which patients with NMIBC receive neoadjuvant CVA21 or low dose Mitomycin C plus CVA21 prior to routine surgical removal (TURBT). The Phase I/II CANON (CAVATAK in NONMuscle invasive bladder cancer: NCT02316171) study investigating the tolerance of multiple escalating intravesicular doses of CVA21 has shown general tolerance of the intravesicular CVA21 treatment. Serial photography has identified possible evidence of viral induced surface haemorrhage and inflammation of the tumour micro-environment. Potential multicycle virus replication within the tumour was highlighted by detection of secondary viral load peaks in the urine as evidenced by RTPCR and infectious TCID50 assays.

## Conclusions

TURBT tissue analysis displayed marked tumour specific cytoplasmic viral protein expression and widespread evidence of viral-induced apoptotic cell death.

